# A novel double-negative feedback loop between miR-489 and the HER2-SHP2-MAPK signaling axis regulates breast cancer cell proliferation and tumor growth

**DOI:** 10.18632/oncotarget.7577

**Published:** 2016-02-22

**Authors:** Yogin Patel, Nirav Shah, Ji Shin Lee, Eleni Markoutsa, Chunfa Jie, Shou Liu, Rachel Botbyl, David Reisman, Peisheng Xu, Hexin Chen

**Affiliations:** ^1^ Department of Biological Science, University of South Carolina, Columbia, SC, USA; ^2^ Center for Colon Cancer Research, University of South Carolina, Columbia, SC, USA; ^3^ Department of Surgery, Chonnam National University, Gwangju, Republic of Korea; ^4^ Department of Drug Discovery and Biomedical Sciences, South Carolina College of Pharmacy, University of South Carolina, Columbia, SC, USA; ^5^ Master of Science in Biomedical Sciences Program, Des Moines University, Des Moines, IA, USA

**Keywords:** microRNA, miR-489, HER2, breast cancer, tumor suppressor

## Abstract

Human epidermal growth factor receptor 2 (HER2 or ErBb2) is a receptor tyrosine kinase overexpressed in 20-30% of breast cancers and associated with poor prognosis and outcome. Dysregulation of several microRNAs (miRNAs) plays a key role in breast cancer progression and metastasis. In this study, we screened and identified miRNAs dysregualted in HER2-positive breast cancer cells. Our molecular study demonstrated that miR-489 was specifically downregulated by the HER2-downstream signaling, especially through the MAPK pathway. Restoration or overexpression of miR-489 in HER2-positive breast cancer cells significantly inhibited cell growth *in vitro* and decreased the tumorigenecity and tumor growth in xenograft mice. Mechanistically, we found that overexpression of miR-489 led to the decreased levels of HER2 and SHP2 and thus attenuated HER2-downstream signaling. Furthermore, we for the first time demonstrated that HER2 is a direct target of miR-489 and therefore HER2-SHP2-MAPK and miR-489 signaling pathways form a mutually inhibitory loop. Using quantitative real-time PCR analysis and Fluorescent *in situ* hybridization technique (FISH), we found that miR-489 was expressed at significantly lower level in tumor tissues compared to the adjacent normal tissues. Downregulation of miR-489 in breast cancers was associated with aggressive tumor phenotypes. Overall, our results define a double-negative feedback loop involving miR-489 and the HER2-SHP2-MAPK signaling axis that can regulate breast cancer cell proliferation and tumor progression and might have therapeutic relevance for HER2-positive breast cancer.

## INTRODUCTION

Breast cancer is a heterogeneous disease with several subtypes identified by unique molecular signatures [1–3]. Nearly 30% of total breast cancer patients overexpress human epidermal growth factor receptor 2 (ErbB2 or HER2) [4–7]. It is well documented in the literature that overexpression of HER2 promotes aggressive tumor phenotype, increases metastasis and decreases overall survival of patients [3, 6, 8]. Trastuzumab, a monoclonal antibody to HER2, accrues significant clinical benefit in the metastatic and adjuvant settings. However, some patients suffer disease recurrence despite adjuvant trastuzumab therapy, and many patients with metastatic disease do not respond to therapy or develop refractory disease within 1 year of treatment [4, 5]. Thus, identification of specific molecular factors, which can regulate HER2 signaling is critical for more targeted and efficient therapy against HER2 positive breast cancer. microRNAs are small (18-25 nucleotide) long non-coding, single stranded RNAs, which regulate the expression of various genes at post-transcriptional level mostly by binding to the partially complementary site on 3′UTR region of target mRNA [3, 9, 10]. It has also been implicated that HER2 regulates the expression of specific miRNAs to promote cell proliferation and tumorigenesis. In a micro array study, Mattie *et al.* found that several miRNAs are down-regulated in HER2 positive tumors compare to the HER2 negative tumors. Down-regulation of miR-205 by HER2 is shown to enhance tumorigenesis in breast cancer. [11]. A recent study has found that hyper-methylation of miR-200b promoter is associated with higher HER2 expression [12]. Moreover, aberrant expression of specific miRNAs by HER2 leads to the enhanced resistance to chemotherapeutic drugs [13–16]. However, it still remains largely unknown how HER2 promotes tumor progression via regulation of specific microRNAs.

A few recent studies have shown that miR-489 plays an important role in both development and tumorigenesis. Cheung *et al.* has shown that the miR-489 pathway is essential for the maintenance of the quiescent state of muscle stem cells [17]. In addition, miR-489 seems to play a tumor suppressive role in a few different types of cancers. The expression of miR-489 is downregulated in hypopharyngeal squamous cell carcinoma (HSCC), non-small cell lung cancer (NSCLC) and in breast cancer [18, 19]. Overexpression of miR-489 inhibited cell growth and invasion and epithelial-to-mesenchymal transition (EMT) properties by targeting several genes including *Shp2*, *Smad3*, *Akt3* and *Suz12*. In addition, loss of miR-489 expression confer tumor cells resistance to chemotherapeutic drugs [20]. Overall, these data suggest that dysregulation of miR-489 may contribute to tumor development and affect the sensitivity to anti-cancer drugs by regulating different target genes in a context-dependent manner.

In this study, we identified miR-489 as one of the candidate miRNAs whose expression was downregulated by HER2 downstream MAPK signaling and for the first time showed that miR-489 directly binds to the 3′-UTR of *HER2* mRNA and down-regulates its expression. We also confirmed that miR-489 can target another *HER2* downstream gene *Shp2* in breast cancer cells. Therefore, the HER2-SHP2-MAPK and miR-489 signaling pathways form a double negative feedback loop which regulates breast cancer cell proliferation both *in vitro* and *in vivo*. Moreover, our clinical data analysis indicated that miR-489 expression was significantly downregulated in tumor tissues compared to the normal breast tissues from same patients. The close association of miR-489 expression with clinical parameters implicates miR-489 can be a new prognostic marker and potential therapeutic target for breast cancer.

## RESULTS

### Screening and identification of HER2-regulated miRNAs in breast cancer cells

**Figure 1:**
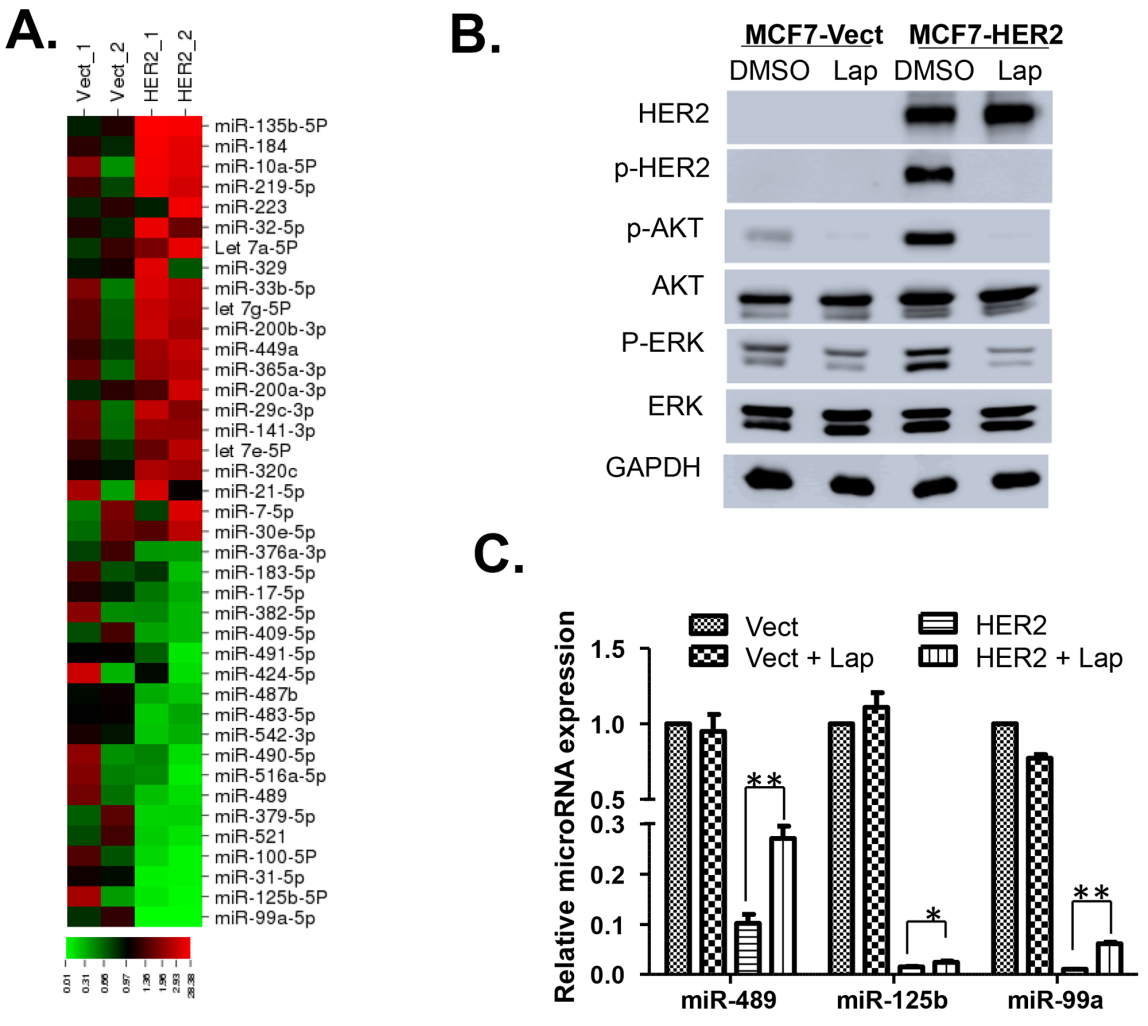
Screening of miRNAs differentially expressed in MCF7 Vector and HER2 cells **A.** Out of total ~200 miRNA screened for differential expression between MCF-7 vect and MCF-7 HER2 cells, ~40 miRs showed significant difference in their expression as shown in the schematic heat-map representation. Expression levels of each miRNA was quantified by qRT-PCR analysis from three independent experiments. Heatmap colors are indicative of miRNA expression as shown in the color key. **B.** Western blot analysis of HER2, p-HER2, p-AKT, Total AKT, p-ERK, Total ERK and GAPDH from total protein lysates isolated from MCF7 Vect and HER2 cells treated with 2μM Lapatinib (HER2 activation inhibitor). **C**. qRT-PCR analysis of selective downregulatedmiRNAs whose expression was rescued by lapatinib treatment of MCF-HER2 cells. All data are representative of three independent experiments. * p value < 0.05; ** p value < 0.01.

To identify specific miRNAs that may be involved in promotion of breast cancer progression, we screened expression profiles of several (~200) miRNA between HER2 over-expressing and control MCF7 cells using quantitative RT-PCR (qRT-PCR) analysis (Supplementary Table S1). We found that 21 of the dysregulated miRNAs were significantly upregulated, whereas 19 were down regulated in MCF7 HER2 cells compared to the MCF7 Vect cells (Figure 1A). Many of these upregulated miRNAs were known oncomiRs in breast cancer, such as miR-200, miR-141 and miR-223 [21–23], Also, many of down-regulated miRNAs in MCF7 HER2 cells were tumor suppressors, such as miR-125b, miR-31 and miR-99a [24–26]. To determine specific miRNAs truly driven by HER2 signaling, we blocked the HER2 kinase activity by treating MCF7 vect and MCF7 HER2 cells with EGFR/HER2 dual inhibitor lapatinib for 48 h. Lapatinib treatment as expected, resulted in the almost complete blockade of HER2, AKT and Mitogen activated protein kinase (MAPK) phosphorylation (Figure 1B), a readout of lapatinib inhibition of HER2 signaling. We then performed the qRT-PCR using the RNA from lapatinib treated vect and HER2 cells to identify miRNAs which restore their expression by inhibition of HER2 signaling (data not shown). Our qRT-PCR data demonstrated that miR-489, miR-125b and miR-99a at least partially restored their expression profile after inhibition of HER2 phosphorylation (Figure 1C). We decided to focus further on the function of miR-489 given that very little is known about its function in breast cancer.

### HER2 negatively regulates miR-489 mainly via the MAPK pathway

**Figure 2:**
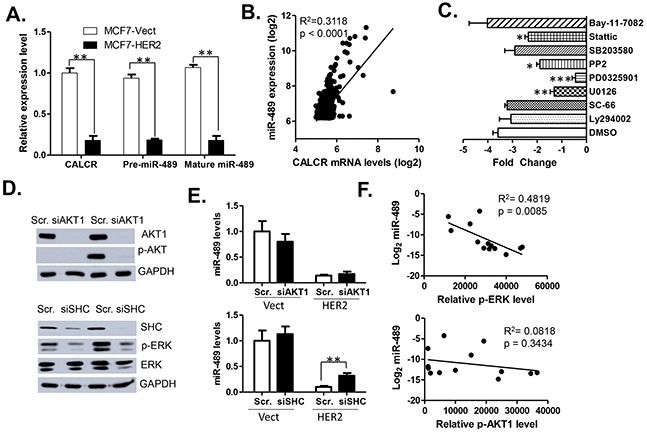
Expression of miR-489 is downregulated at transcriptional level by HER2 signaling **A.** Real-time PCR analysis ofcalcitonin receptor (CALCR) mRNA, pre-miR-489 and mature miR-489 RNA levels in MCF7-HER2 and MCF7-vect cells. **B.** Correlation between CALCR mRNA and mature miR-489 in clinical breast cancer tissue. The linear correlations between CALCR and miR-489 ligand expression in primary breast cancer tissues were evaluated with Pearson correlation coefficient analysisusing public dataset [51]. **C.** Effects of HER2-downstream signaling inhibitor treatments on the expression of miR-489 in MCF-7 HER2 cells. MCF-7 HER2 cells were treated with each of the following inhibitors Bay-11 7082 (2μM), Stattic (2μM), SB-203580 (10 μM), PP2 (10 μM), PD0325901 (10 μM), U0126 (10 μM), SC-66 (2μM), LY294002 (10 μM) for 24 h. **D.** Western blot showing the effect of siAKT on the expression of AKT, pAKT and effect of siSHC on SHC, p-ERK and ERK expression. **E.** Effects of siRNA-mediated blockage of AKT and MAPK signaling on the expression of miR-489. **, p<0.01; *, p<0.05. **F.** Pearson analysis of theCorrelation between p-AKT or p-ERK1/2 and mature miR-489 in breast cancer lines.

The miR-489 gene sequence is located in the intronic region of calcitonin receptor (CALCR) gene (Supplementary Figure S1). To find whether HER2 affects the expression of miR-489 at transcriptional level, we quantified the expression levels of mature miR-489, pre-miR-489 and CALCR mRNA in MCF7 vect and HER2 cells using qRT-PCR (Figure 2A). Our data demonstrated that expression of CALCR mRNA, pre-miR-489 and mature miR-489 are down regulated to a similar extent (Figure 2A), suggesting that expression of miR-489 may be controlled at the transcription level. The strong positive correlation between the expression levels of CALCR and miR-489 in clinical samples further confirmed this conclusion (Figure 2B).

Next, to determine the role of a particular signaling pathway in miR-489 transcriptional regulation, MCF7 HER2 cells were treated with the HER2-downstream signaling pathways inhibitors targeting NF-κB (Bay-11-7082), PI3K (LY294002), AKT1 (SC-66), MEK (U0126), MAPK (PD0325901), SRC (PP2), p38 (SB-203580), and STAT3 (Stattic) signaling. The qRT-PCR indicated that miR-489 levels were most significantly rescued by two inhibitors, both targeting the MEK-ERK signaling (Figure 2C). These results led us to believe that MEK-ERK signaling might be crucial in controlling the miR-489 transcription, although STAT3 and SRC pathways may also be involved in its regulation.

To further validate that miR-489 might be regulated by MEK-ERK signaling possibly through HER2 down-regulation, we treated cells with si-AKT1 and si-SHC to block two downstream effectors of HER2 signaling. As shown in the Figure 2D, treatment of si-AKT1 or si-SHC resulted in decreased AKT1 expression and decreased activation of p-ERK respectively. However, expression of miR-489 was rescued only by si-SHC knockdown and not by AKT1 knockdown. Furthermore, to understand triggering of which pathway results in miR-489 down regulation, we isolated proteins from 13 breast cancer cell lines and performed western blotting for p-ERK and pAKT (Supplementary Figure S2). Protein expression data was quantified and correlated with the expression of miR-489 in each cell line. The statistical data clearly demonstrated that p-ERK, not p-AKT, was negatively correlated with the levels of miR-489 in all 13 breast cancer cell lines (Figure 2F). Overall, these results clearly demonstrated that miR-489 expression is transcriptionally regulated mainly by the HER2-driven MAPK downstream signaling.

### Overexpression of miR-489 inhibits cell growth *in vitro*

**Figure 3:**
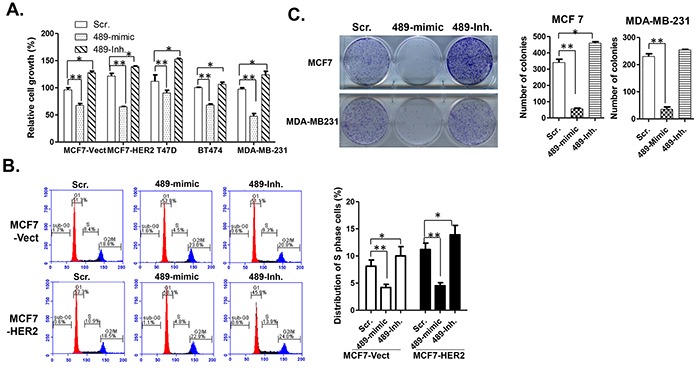
Overexpression of miR-489 in breast cancer cells inhibits cell proliferation **A.** MTT cell viability assay showing the rate of cell proliferation of MCF7-Vect and HER2, BT-474, T47D and MDA-MB-231 cells transfected with miR-489 mimic or inhibitor for 72 h. **B.** Cell cycle analysis of cells transfected with miR-489 mimic and inhibitor using FACS. Representative FACS profiles and average percentages of S phase cells were shown in the figure. **C.** Colony formation assay on MCF7 and MDA-MB231 cells transfected with either scramble, mimic miR-489 or inhibitor mir-489 * p<0.05, ** p value < 0.01.

To explore the biological function of miR-489 in breast cancer, we transiently transfected breast cancer cells with miR-489 mimic or inhibitor and measured cell growth by MTT assay. Our results clearly indicated that miR-489 over expression inhibited cell proliferation in all of the four tested breast cancer cell lines and conversely, knockdown of miR-489 increased cell proliferation (Figure 3A). However, we didn't observe significant changes in cellular morphology and the number of floating cells (Supplementary Figure S3). To test the possibility that miR-489 may regulate cell cycle progression, we examined the cell cycle profiles by FACS analysis. Indeed, we observed a dramatic decrease or increase in the S phase population of miR-489 mimic or miR-489 inhibitor transfected cells respectively (Figure 3B). Consistently, overexpression of miR-489 appeared not to induce apoptosis, which is evident by slight changes in the Sub-G_0_ cell populations. A similar trend was observed in both MCF7-vect and MCF7-HER2 cells although changes were more dramatic in MCF7-HER2 cells. Next, to examine whether miR-489 inhibits colony formation ability of cancer cells, both MCF7 and MDA-MB-231 cells transfected with either the scramble, mimic or miR-489 inhibitor were used to perform colony formation assay. Our results clearly demonstrated that cells transfected with miR-489-mimic yielded significantly fewer colonies compared to the cells transfected with scramble miRNA (Figure 3C). Conversely, cells transfected with inhibitor of miR-489, as predicted had opposite effect and resulted in forming more colonies than the cells transfected with scramble miRNA. Taken together, increased expression of miR-489 in breast cancer cells resulted into inhibited cell proliferation and transformation.

### miR-489 regulates HER2 signaling pathway by directly targeting *HER2* and its downstream gene *Shp2*

**Figure 4:**
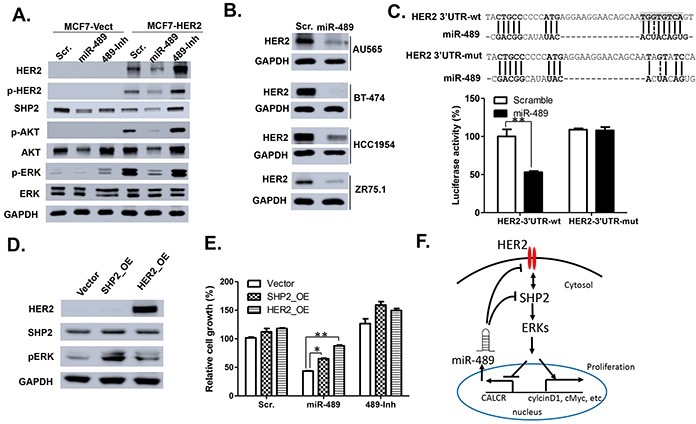
miR-489 targets HER2 signaling pathway by directly binding the 3′ UTR of HER2 **A.** Effects of miR-489 and inhibitor treatment on HER2-downstream signaling. Western blot analysis was performed after MCF-7 vector/HER2 cells were transfected for 72 hrs. **B.** Western blot analysis of other breast cancer cell lines treated with miR-489 mimic also showed reduction in HER2 expression. **C.** A schematic representation of the HER2 mRNA with putative miR-489 binding site in the 3′ UTR, where the seed region is highlighted. MCF7 HER2 cells were co-transfected with either of these vectors with miR-489 expressing vector or empty vector and renilla expressing vector for 48 h. Firefly luciferase was measured for each condition and normalized with *renilla* luciferase. Normalized luciferase activity was compared with WT-3′UTR and Mutant 3′ UTR of HER2. **, p value < 0.01. **D.** Western blot showing expression of SHP2 and HER2 in SHP2 and HER2 OE MDA-MB231 cells. **E.** MTT assay showing relative cell survival of vector control, SHP2 OE or HER2 OE cells transfected with scramble, mimic miR-489 or inhibitor miR-489 **, p<0.01; *, p<0.05. **F.** General feedback loop model proposed by the study, where HER2 signaling through SHP2 and ERK promotes cell proliferation and inhibits miR-489 expression, whereas miR-489 downregulates both HER2 and SHP2 directly to inhibit cell proliferation.

According to the miRwalk pathway target prediction, HER2 pathway can be potentially targeted by miR-489. To verify whether miR-489 has any effect on HER2 signaling, we transfected MCF7 Vect and HER2 cells with mimic or inhibitor of miR-489 and total protein was isolated after 72 h. Western blotting analysis was performed to examine the HER2 signaling in the transfected cells (Figure 4A). Our results indicated that miR-489 mimic dramatically impaired HER2 signaling as evident by reduced HER2, phospho- HER2, SHP2, phospho-AKT and phospho-ERK (Figure 4A). Conversely, miR-489 inhibitor transfected cells exhibited a reversed effect on HER2 signaling compared to miR-489 mimic. Moreover, we also observed that expression of total HER2 and SHP2 were reduced in miR-489 mimic transfected cells and elevated in the miR-489 inhibitor transfected cells (Figure 4A). Previous studies showed that miR-489 can downregulate SHP2 expression in hypopharyngeal squamous cell carcinomas by directly binding to its 3′UTR[18]. We confirmed that overexpression of miR-489 reduced SHP2 expression in breast cancer cell lines at both mRNA and protein levels (Supplementary Figure S4). To examine the similar effect of mimic-489 treatment on the expression of HER2, other breast cancer cell lines, AU-565, BT-474, HCC-1954 and ZR-75-1 cells were treated with mimic-489 for 72 hours to isolate total protein and RNA. Our western blotting data demonstrated that the expression of HER2 is decreased in all tested cell types treated with mimic-489 (Figure 4B). Together, our results strongly suggested that miR-489 may directly target HER2 and SHP2 in breast cancer cells and regulate its expression. By searching through 3′UTR sequence of HER2, we identified a potential partially complementary miR-489 binding site. To demonstrate that this site is functional, we performed the dual luciferase assay using reporter constructs containing ~400 bp long wt 3′UTR or mutant 3′ UTR of *HER2*. Our data showed that in the presence of miR-489, luciferase activity of wt *HER2* 3′UTR and not the mutant *HER2* 3′UTR is significantly reduced (Figure 4C). These results clearly demonstrated that miR-489 inhibits HER2 expression by directly binding to its 3′UTR region.

Previous studies have validated one of the downstream effector of HER2 signaling SHP-2 as the direct target of miR-489 [18, 27]. SHP-2 is known to affect ERK signaling [28, 29]. Since p-ERK levels were also inversely correlated with the expression of miR-489, we hypothesized that miR-489 affects ERK signaling by downregulating the expression of HER2 and SHP2. Using a lentiviral system, we constructed MDA-MB-231 cells over-expressing (OE) either HER2 or SHP2 (Figure 4D). Also, level of p-ERK was increased in both SHP2 and HER2 OE cells as shown in western blot (Figure 4D). To demonstrate the effect of SHP2 or HER2 OE on cell survival against miR-489, SHP2 and HER2-overexpressing MDA-MB-231 cells were transfected with either mimic or inhibitor of miR-489. Our MTT data indicated that both SHP2 and HER2 overexpression led to the increased cell survival significantly when compared to the vector control cells in the presence of miR-489 mimic (Figure 4E). These results overall allow us to create a double feedback loop model where HER2 and SHP2 activates ERK signaling which results in the inhibition of miR-489 expression, while miR-489 targets both SHP2 and HER2 simultaneously to affect the ERK signaling and therefore decrease the cell proliferation (Figure 4F).

### Over expression of miR-489 inhibits tumor growth *in vivo*

**Figure 5:**
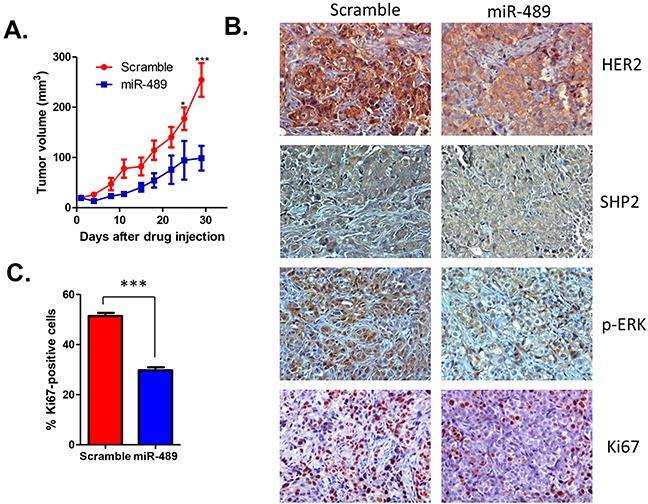
Overexpression of miR-489 inhibits tumor growth in vivo **A.** Nanoparticle-delivered miR-489 inhibits tumor growth. After the tumors were palpable, all 7 tumor sites were injected with miR-489 or scramble miRNA encapsulated in nanoparticle every three times. The treatment starting day was referred to as ‘Day zero’ in the figure. **B.** IHC analysis of cells in tumors treated with nanoparticle-delivered miR-489 or scrambled miRNA for HER2, SHP2, p-ERK and Ki-67. **C.** Graph showing number of Ki-67 positive cells intumors treated with nanoparticle-delivered miR-489 or scrambled miRNA. ***, p value < 0.001.

Since miR-489 inhibited cell proliferation and decreased transforming capacity of cells to form colonies *in vitro* (Figure 3C), we wanted to assess its ability to inhibit tumor growth *in vivo*. To explore the possibility to use miR-489 for therapy, we developed a nanoparticle delivery system to deliver miR-489 into tumor cells. The nanoparticle packaged with miR-489 showed similar size distribution as control nanoparticles (Supplementary Figure S4A). Treatment of cells with increased concentration of nanoparticle resulted in a decrease in HER2 expression levels in a dose-dependent manner (Supplementary Figure S4B), indicating that nanoparticles can effectively deliver miR-489 into tumor cells. In this xenograft experiment, athymic nude mice were injected with ~2 million HCC1954 cells and monitored for tumor growth randomly assigned in 2 groups. After tumors were palpable, both groups one and two were given the intra-tumoral injection of nanoparticles packaged with scramble miRNA or mimic-489 respectively every 3 days. Our tumor volume data indicates that in the last two time points tumor sizes of the mimic-489 injected group of mice were significantly (p<0.001) smaller than the scramble injected group of mice. Moreover, our IHC data also revealed that HER2 and SHP2 expression was lower in miR-489 injected tumors compared to the tumors injected with scramble (Figure 5B). Our IHC data indicated that p-ERK level was reduced in miR-489 injected tumors compared to the tumors injected with scramble. Also, number of ki-67-positive cells was significantly lower in miR-489 injected tumors when compared to the scramble injected tumors (Figure 5C). Together, these data demonstrated that miR-489 delivered through nanoparticles inhibits tumor growth in xenografts by decreasing cell proliferation at least partially by blocking the HER2-, SHP2-MAPK signaling axis.

### Low expression of miR-489 in primary breast cancer is associated with aggressive phenotypes and poor clinical outcomes

**Figure 6:**
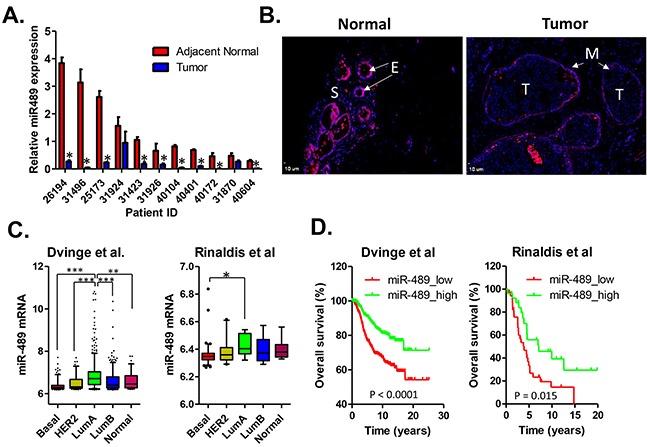
Expression status of miR-489 in primary breast cancer tissues **A.** Real-time RT-PCR analysis of miR-489 in breast cancer tumor tissues and their adjacent normal tissues from 11 breast cancer patients. *, p value < 0.05. **B.** FISH analysis of miR-489 expression in normal breast tissue and adjacent tumor tissues. E, normal epithelial cells; S, stromal cells; M, myoepithelial cells; T, tumor cells. **C.** Relative expression levels of miR-489in breast cancer subtypes were compared using the one-way ANOVA analysis. The microarray data was extracted from previous publication [51, 52]. *, p value < 0.05; **, p value < 0.01; ***, p value < 0.001. **D.** Expression status of miR-489 predicts clinical outcome. Patient survival was estimated using the Kaplan-Meier method and compared with log-rank tests. The Y axis represents the probability of overall survival.

To explore the clinical relevance of miR-489 expression in breast cancer, we performed quantitative RT-PCR analysis of cDNAs generated from 11 pairs of breast cancer and their corresponding adjacent normal tissue. Compared to the adjacent normal tissues, primary breast cancers expressed lower levels in 9 out of 11 pairs of samples (Figure 6A). To further validate that loss of miR-489 expression in breast cancer epithelial cells, we did *in situ* fluorescent hybridization on breast cancer tissue and adjacent normal tissues. High levels of miR-489 expression were detected in normal epithelial cells and occasionally myoepithelial cells, however, the staining signal intensities were weak in the stromal and tumor areas (Figure 6B). Furthermore, we analyzed the correlation between miR-489 expression level and other clinical parameters including overall survival, HER2 status, metastasis, grade and stages (Supplementary Table S2). We found that there is an inverse correlation between the expression of miR-489 and HER2 in clinical samples as indicated by our *in vitro* data. Loss of miR-489 expression is especially associated with tumor in higher grades and higher stages (Supplementary Table S2). We also found that the expression levels of miR-489 tend to be lower in HER2-positive and basal subtypes compared to both luminal and normal-like subtypes of breast cancer (Figure 6C). Given that the expression statuses of miR-489 tend to be associated with the aggressive subtypes of breast cancers, we performed the Kaplan-Meier survival analysis to further evaluate the prognostic value. As expected, patients with low miR-489 expression had a relatively poor overall survival than those with high miR-489 expression (Figure 6D). In addition, multivariate analysis revealed that miR-489 expression was an independent prognostic factor to predict overall patient survival (Table 1). Together, our results support that miR-489 can be serve as a prognostic marker in breast cancer and loss of miR-489 in breast cancer may contribute to tumor progression.

**Table 1:** Multivariate cox proportional hazard regression model of the risk of overall survival according to miR-489 expression adjusted for clinic-pathological factors

Variables	HR	CI (95%)	P value
Tumor Size (≤2cm/>2cm)	1.016	1.006-1.026	<0.001
Tumor Grade			
Grade (I/II)	1.802	0.902-3.601	0.095
Grade (I/III)	2.422	1.221-4.804	0.011
Tumor stage			
Stage (I/II)	1.205	0.804-1.807	0.367
Stage (I/III, IV)	1.860	1.040-3.326	0.036
HER2 status (negative/positive)	1.691	1.232-2.323	0.001
Lymph node (negative/positive)	1.769	1.265-2.476	<0.001
miR-489 (low/high)	0.606	0.433-0.850	0.004

HR, hazard ratio; CI, confidence interval.

## DISCUSSION

Deregulated miRNA expression can promote tumorigenesis and contribute to a variety of cancer phenotypes including uncontrolled cell proliferation, invasion and metastasis. HER2-overexpressing breast cancers are generally associated with aggressive phenotypes and poor prognosis. However, how miRNAs are involved in HER2 signaling pathways are not well understood. There are several reports on HER2-regulated miRNAs in breast cancers [30–33]. A comparison of our data with these published expression signatures revealed several miRNAs previously found to be associated with HER2 such as miR-125, miR-99a and miR-21 [30, 33, 34].

Among many deregulated miRNAs in HER2-overexpressing breast cancer cells, miR-489 expression may be directly regulated by HER2 signaling since treatment with HER2/EGFR dual inhibitor lapatibnib can significantly reverse miR-489 expression status in HER2-positive breast cancer cells. Loss of miR-489 expression in HER2-positive breast cancer cells suggests that miR-489 might have tumor suppressive function. Previously, it was already known that miR-489 functions as a tumor suppressor gene in hypopharygeal squamous cell carcinoma (HSCC)[18]. In this study, a screening for miRNAs that inhibited cancer cell proliferation was performed. Among different miRNAs, miR-489 stood out, as its overexpression inhibited cell growth in all cell lines examined [18]. Consistently, we show that restoration of miR-489 in HER2-overexpressing breast cancer cells strongly inhibits cell proliferation both *in vitro* and *in vivo*. Unlike many tumor suppressors that normally induce cell cycle arrest and apoptosis, miR-489 dramatically reduced ki-67 positive population, but didn't induce apoptosis. Conversely, transfection of miR-489 inhibitor can further increase cell proliferation rate which is evidenced by increase cell population in S phase. This data indicated that miR-489 may be directly or indirectly involved in regulating cell cycle progression.

In a previous screening for microRNAs, which are essential for HER2 positive breast cancer cell growth, miR-489 has been identified as one of the potential candidates to attenuate HER2 signaling, but the underlying mechanism remains unknown [35]. In this study, we for the first time demonstrated that HER2 is a bona fide target of miR-489 by the following evidences. First, overexpression of miR489 results in down-regulation of HER2 in several breast cancer cell lines. In a complementary experiment, transient transfection of miR-489 inhibitor can increase the protein levels of HER2. Secondly, a putative miR-489 binding site is identified in the 3′UTR of HER2 mRNA. Thirdly, the expression levels of miR-489 is inversely correlated with HER2 expression status in clinical breast cancer samples. Identification of HER2 as a new target in breast cancer cells may shed light on the function of miR-489 in breast tumorigenesis. It should also be kept in mind that besides HER2, miR-489 may target many other target genes as well. For example, miR-489 has been shown to directly target other oncogenes such as *Shp2* in HSCC [18], SMAD3 in breast cancer [19], AKT3 in ovarian cancer [36] and Dek in mouse muscle stem cells [17, 36]. The *Shp2* gene is unusual in that it promotes the activation of RAS—MAPK signaling pathway downstream of EGFR family receptors [37, 38]. We have also confirmed that miR-489 can directly downregulate SHP2 expression in breast cancer cells. One could envisage that miR-489 can dramatically modulate HER2 signaling networks via simultaneously targeting multiple downstream genes during breast carcinogenesis.

Understanding the signaling cross-talk between miR-489 and HER2 indicate that the HER2-SHP2-MAPK signaling axis and miR-489 form a double-negative regulation loop to regulate cell proliferation in breast cancer (Figure 4F). Double-negative feedback loop involving microRNAs and their targets have been observed previously [39–42]. As a general model, the double-negative feedback loop is advantageous in allowing the system to remain reversible. This feature is evident in the previous observations that the switching ability between epithelial and mesenchymal states can be regulated by the ZEB/miR-200 feedback loop [41]. Here, it is likely that the HER2-SHP2-MAPK/miR-489 feedback loop is involved in regulation of quiescence/proliferation phenotypical transition in cancer cells. HER2 expression is associated with higher cell proliferation, whereas miR-489 expression may be associated with slow proliferation or quiescent status [17]. It remains unknown whether the expression ratio of HER2/miR-489 determines the overall cell proliferation rate of a tumor or tumor heterogeneity with mixed cell population with high/slow proliferation rates. Given that a double-negative feedback loop generally allows the maintenance of bi-stable states, we tend to believe that the ratio of HER2/miR-489 is critical for generation of tumor heterogeneity and determination of tumor behaviors.

Although our clinical data shows that miR-489 expression is lower in HER2-positive tumors, its expression can be downregulated in many human breast cancers independent of their HER2 expression status. Because miR-489 is mainly regulated via the MEK-ERK pathway which can be activated by many overexpressing oncogenes or receptors or cytokines. For example, expression of multiple members of the epidermal growth factor receptor family (HER1, HER2, or HER3) and estrogen receptor is present in most breast cancer cells, and these receptors have all been shown to activate the MEK-ERK pathway[43–45]. This may explain why miR-489 is broadly down-regulated in breast cancer cells compared to their adjacent normal tissues, especially in HER2-positive and basal subtypes of breast cancers. It was worth mentioning that hyperactivation of ERK1/2/MAPK signaling occurring downstream of EGFR or HER2 in breast cancer can induce loss of ER expression leading to establishment of ER-negative phenotype [46]. Cancers with hyperactivation of ERK1/2/MAPK signature are associated with adverse clinical features and especially enriched for basal-like and HER2-positive subtypes [47]. Consistently, our data showed that the expression levels of miR-489 are lower in basal-like and HER2-positive subtypes compared to luminal subtypes of breast cancers. Given that the expression levels of miR-489 is strongly correlated with tumor aggressive phenotypes, it raises the possibility that targeting miR-489 as a therapy may be of benefit not just in HER2/*neu*-overexpressing tumors, but also in a broader subsets of patients.

## MATERIALS AND METHODS

### Cell lines and reagents

MCF7 vect and MCF7 HER2 cell lines were kindly provided by Dr. Rachel Schiff (Baylor College of Medicine) and were grown in DMEM supplemented with 10% FBS and 5 μg/ml insulin. Other breast cancer cell lines AU-565, BT-474, HCC1954, T47D, ZR-75-1, HCC1569 and ZR-75-3 were purchased from ATCC and cultured in RPMI-1640 containing 10% FBS. All cells were cultured at 37°C in a humidified incubator containing 5% CO_2_. MDA-MB-468, MDA-MB -231, MDA-MB -435, SK-BR-3 and MDA-MB -453 were purchased from ATCC and cultured in DMEM containing 10% FBS. All cells were cultured at 37°C in a humidified incubator containing 5% CO_2._ DOTAP (N-[1-(2, 3-Dioleoyloxy) propyl]-N, N, N-trimethylammonium methyl-sulfate) and cholesterol were purchased from Avanti Polar Lipids. Protamine sulfate was from Sigma-Aldrich and Sodium hyaluronate was purchased from Lifecore biomedical.

### Plasmid constructs

A 650 bp long 3′ UTR of human HER2 was amplified from genomic DNA and cloned into the pGL3-promoter vector at the same *Xba* I and *Bam*H I sites. For the mutation analysis putative miR-489 target site within 3′UTR of *HER2* was mutated by Phusion site directed mutagenesis kit (Thermofisher Cat# F-541). All sequences were verified by direct sequencing of the plasmids.

### Stable cell line generation

293T cells were transfected with 3μg of SHP2 (Addgene #8329)/HER2 plasmid (provided through the courtesy of Emily Wang at the Institute of City of Hope) [48], 3μg of VSVG plasmid and 2.5μg of ECO plasmid using 17μl of Lipofectamine2000 for 48 hours. After 48 hours, supernatants were collected and spin down at 10,000 RPM at 4°C to discard any cell debris. MDA-MB -231 cells were infected with SHP2 and HER2 lentivirus for 48 hours. Infected cells must be puromycin resistant and therefore cells were treated with puromycin for one week.

### Preparation of miR-489-delivering nanoparticle

Liposomes were prepared as described elsewhere with some modifications [49, 50]. Briefly small unilamellar liposomes consisting of DOTAP and cholesterol were prepared by thin film hydration method. The film was hydrated with nuclease free water and sonicated in a bath type sonicator for 5 min followed by extrusion through 200 and 50 nm membrane filters. The total lipid concentration of the liposomes was fixed at 28.6 mM (20 mg/ml). For detailed preparation of miRNA-nanoparticles see supplementary methods.

### Clinical tissues and fluorescent *in situ* hybridization (FISH)

Human breast cancer tissue samples were obtained through the South Carolina Tissue Bank with approval from the Institutional Review Board at the University of South Carolina. Tissue samples were randomly collected from patients who were diagnosed with invasive breast ductal carcinoma between 2003 and 2007. The tissue samples from breast cancer patients who underwent surgery in 2008 were provided by the Chonnam National University Hwasun Hospital National Biobank of Korea, a member of the National Biobank of Korea, which is supported by the Ministry of Health, Welfare and Family Affairs.

Tissue sections were dewaxed and rehydrated with phosphate buffered saline (PBS) and fixed using 4% paraformaldehyde (PFA). Next, tissue sections were treated with proteinase K (50μg/μl, life technology) for 15 min at 37°C. Acetylation of tissue sections was performed with 0.1M triethanolamine/0.25% acetic anhydride for 5 min. Sections were washed once with PBS. Before proceeding with hybridization step, Hybridization buffer was prepared by mixing 250 μl of formamide, 250 μl of 20x SSC, 50x Denhard't solution (life technology), 12.5 μl of t-RNA (Roche), 2.5 μl of Herring Sperm DNA (Promega D1815), 0.02 gm of blocking reagent (Roche) and 30 μl of RVC (Fisher) in 1 ml. Sections were pre- hybridized with hybridization buffer for 2 h at room temperature.

LNA-substituted DNA oligonucleotide probe for miR-489 was obtained from Exiqon (Cat#38599-01) labelled with digoxigenin at the 5′ terminus. Probes were denatured at 95°C for 5 min, then chilled on ice for 5 min. Total of 100 μl diluted probe was added to cover tissue area. Slides were kept in the hybridization chamber and incubated in oven at 55°C overnight. Next day, sections were washed once with 5x SSC for 7 min and twice each with 1x SSC (7 min) and 0.2x SSC (7 min) at 57°C, followed by two 7 min washes with 0.2x SSC at room temperature and final wash with 1x PBS. Sections were blocked with blocking reagent (life technology: E-6604) for 1 hour at room temperature, followed by incubation with anti-DIG AP labelled primary antibodies for 1 h at room temperature (Roche: 11093274910). Sections were washed with 1x PBS for 5 min and developed for microscopic visualization using ELF-97 kit from Life technology.

### Statistical analyses

The statistical analyses were conducted with R and GraphPad software packages (GraphPad). A Student t test or ANOVA test was used for comparison of quantitative data. The linear correlations between CALCR and miR-489 ligand expression in primary breast cancer tissues were evaluated with Pearson correlation coefficient analysis. The clinical effect of the gene expression profiles of miR-489 was further evaluated using two published datasets: Dvinge et al, 1302 breast cancer patients [51], and Rinaldis et al, 181 breast cancer patients [52]. The patient samples were stratified into three equal groups based on the expression levels of miR-489. The two compared groups are the third of patients with the highest expression levels (high) *versus* the third of patients with the lowest expression (low) [53]. Overall survival was estimated using the Kaplan-Meier method and compared with log-rank tests. Univariate regression analysis and multivariate Cox PH regression analysis were performed to demonstrate the correlation between the patient survival and miR-489 while accounting for other potential predictors (covariates). The stepwise Akaike Information Criteria (AIC) method with “both” direction search was used with R step AIC function in the predictor variable selection during the multivariate regression. The selected model was assessed with the overall Goodness-of-fit test. Chi-square tests were made with the Schoenfeld residuals to check the proportional hazard assumption of the selected model. Values of P < 0.05 were considered significant.

### Other methods

Quantitative real-time RT-PCR analysis, Immunohistochemistry staining (IHC), Western Blot analysis, Fluorescence-Activated Cell Sorting (FACS)assay, Luciferase assay and Xenograft experiments were performed using standard protocol [53–55]. See supplementary methods for more details.

## SUPPLEMENTARY MATERIAL AND METHODS, FIGURES, TABLES




