# Supplementary material for: A novel double-negative feedback loop between miR-489 and the HER2-SHP2-MAPK signaling axis regulates breast cancer cell proliferation and tumor growth
Source: Oncotarget. 2016 Feb 22;7(14):18295–308. doi: 10.18632/oncotarget.7577 (PMC4951289; doi:10.18632/oncotarget.7577)
Supplement: Supplementary file 1 [file oncotarget-07-18295-s001.pdf]

SUPPLEMENTARY MATERIAL AND METHODS

Colony formation assay

We transfected mimic-miR-489 and inhibitor-miR-489 in MCF7 and MD-MBA-231 cells and incubated cells for 24 hr [1]. After incubation, we collected all cells. For MCF-7 10000cells/well and for MD-MBA-231 4000cell/well were plated in 6 well plates in triplicates in DMEM media containing 10% Fetal Bovine Serum (FBS). After one week of incubation in 5% CO_2, cells were washed with 1x phosphate-buffered saline (PBS) and fixed using 100% methanol. Once fixed, cells were washed again with 1x PBS and stained with 0.5% crystal violet. All the visible colonies were photographed and manually counted, and the average with standard error of mean was plotted using graphpad prism software.

Western blot quantification

Proteins were isolated from T47D, MCF7, MD-MBA-468, MD-MBA-231, MD-MBA-435, SK-BR-3, MD-MBA-453, AU-565, BT-474, HCC1954, ZR-75-1, HCC1569 and ZR-75-3 by using M-PER protein extraction buffer (Thermo Scientific Cat # 78501) added with 10% protease inhibitor, 2% Sodium Orthovanadate (1mM), 1% Sodium Floride (10mM). Western blot was run as describe previously [1, 2]. Phospho-ERK, Phospho-Akt and GAPDH bands were quantified by using Image Quant TL software. Both Phospho-ERK and Phospho-Akt levels were normalized with GAPDH.

MTT assay

Cell viability was examined by the 3-(4, 5-dimethylthiazol-2-yl)-2,5-diphenyltetrazolium bromide (MTT) assay [1]. Cells were seeded at a density of 4,000 cells/well in triplicate in 96-well plates. Cells transfected with either 0.32µl/well scrambled, miR-489 mimics, or miR-489 Inhibitor by using 0.32µl/well Lipofectamine RNAi and incubated at 37 °C for 72 hr. Cells were seeded at a density of 13,000 cells/well in triplicate in 24-well plates. Cells transfected with either 1µl/well scrambled, miR-489 mimics, or miR-489 Inhibitor by using 1µl/well Lipofectamine RNAi and incubated at 37 °C for 72 hr. MTT assay was performed according to mention above. At the end of the incubation media was removed and formazan precipitates were dissolved by adding 200 µl of DMSO in each well. OD values were measured at both 570 and 630 nm and final values were calculated by subtracting OD_{630} from OD_{570}. These values for each time point were used to plot the growth curve for each clone using graphpad prism.

Cell cycle analysis

Cells were harvested using Accutase, washed with cold PBS and fixed with 70% cold ethanol. Cell pellets were then treated with 100 µg/ml RNase A for 15 min at 37°C and stained using 50 µg/ml Propidium Iodide (PI) for 30 min on ice. Samples were analyzed using BD Accuri flow cytometer [3].

Immunohistochemistry

Immunohistochemistry (IHC) was performed as described previously using 5 µM sections of xenografted tumor fixed in Formalin overnight[4]. Ki-67 (Cat#9027) and HER2 (Cat#2242) were obtained from Cell signaling and antibody for SHP2 was obtained from Protein tech (Cat#20145-1-AP). Images were scored as described previously [4].

Luciferase assay

MCF-7 vect and HER2 cells were seeded at 1.25 x 10^5 cells/well 24 h prior to the transfection in a 24 well plate. Cells were transfected with 500 ng of pGL3 promoter vector containing 3′UTR of *HER2* or mutated 3′UTR, 20 ng of pRL-CMV (Renilla luciferase vector-Promega) and 500 ng of miR-489 pcDNA 3.1 vector or empty pcDNA 3.1 vector. After 48 h post transfection, luciferase activity were measured using Dual-luciferase reporter assay system (Promega Cat# E1980) as per manufacturer′s instructions and normalized to the levels of Renilla luciferase activity [5].

miRNA-nanoparticles preparation

For preparing miRNA-nanoparticles, 50 µl of prepared cationic liposomes 28.6 mM (composed of DOTAP/cholesterol) and 16.6 µl of 2 mg/ml protamine were mixed in a final volume of 100 µl nuclease free water (suspension A). The miRNA liposomes were obtained by thoroughly mixing suspension A with 100 µl of 46 µM miRNA (suspension B). The miRNA-liposomes were allowed to stand at room temperature for 10 min. For HA (Hyaluronic Acid) receptors targeting, miRNA-

nanoparticles were decorated with sodium hyaluronate by adding 27.7 µl of 2 mg/ml sodium hyaluronate solution to suspension B and kept at room temperature for another 10 min before further application. The resulting miRNA-liposomes were used within 20 min for the following experiments or were lyophilized in 30% sucrose for long term storage. The particle size and Zeta potential were measured by dynamic light scattering (DLS) (Malvern Nano-Zs). The sizes of targeted and scramble miRNA liposome were 132.1 ±0.91 and 123.8 ±0.07 nm, respectively (Supplementary Figure S5).

Xenograft experiments

Five to six weeks old athymic female nude mice were purchased from Jackson Laboratories. All mice were handled and maintained under supervision of veterinarian in accordance with institutional guidelines and under a University of South Carolina Institutional Animal Care and Use Committee (IACUC) approved protocol. All mice were subcutaneously injected with 2 x 10^6 HCC1954 in both right and left flank of each mice (n=5/group). The mice tumors were palpated starting day 9 post injection. Tumor volumes were calculated by measuring length and width twice a week for 4 weeks. After 4 weeks of tumor palpation, all mice in the control group were photographed and tumors were extracted after euthanizing all animals. Tumor volumes of each tumor was calculated by modified ellipsoidal formula ($1/2(lxw^2)$). Tumor volumes were plotted using graphpad prism software [1, 4].

Similarly, for nano-particle delivery experiment five to six week old athymic nude female mice were subcutaneously injected with 2 million wt-HCC1954 cells. The day tumors start palpating, each mice were given intratumoral injection of nano-particle containing either scramble or mimic-miR-489 every 3 days for one month (25 µM/treatment). Tumor volumes were measured, calculated and plotted as mentioned above.

REFERENCE

1. Lo PK, Kanojia D, Liu X, Singh UP, Berger FG, Wang Q and Chen H. CD49f and CD61 identify Her2/neu-induced mammary tumor-initiating cells that are potentially derived from luminal progenitors and maintained by the integrin-TGFbeta signaling. Oncogene. 2012; 31:2614-2626.
2. Kanojia D, W. Z, J. Z, C. J, Lo PK, Wang Q and Chen H. Proteomic profiling of cancer stem cells derived from primary tumors of HER2/Neu transgenic mice. Proteomics. 2012; 12:in press.
3. Chen H, Zhang H, Lee J, Liang X, Wu X, Zhu T, Lo P, Zhang X and Sukumar S. HOXA5 acts directly downstream of RARα and contributes to retinoic acid-induced apoptosis and growth inhibition. Cancer Research. 2007; In press.
4. Liu S, Jin K, Hui Y, Fu J, Jie C, Feng S, Reisman D, Wang Q, Fan D, Sukumar S and Chen H. HOXB7 promotes malignant progression by activating the TGFbeta signaling pathway. Cancer Res. 2015; 75:709-719.
5. Chen H, Zhang H, Lee J, Liang X, Wu X, Zhu T, Lo PK, Zhang X and Sukumar S. HOXA5 acts directly downstream of retinoic acid receptor beta and contributes to retinoic acid-induced apoptosis and growth inhibition. Cancer Res. 2007; 67:8007-8013.

SUPPLEMENTARY FIGURES AND TABLES

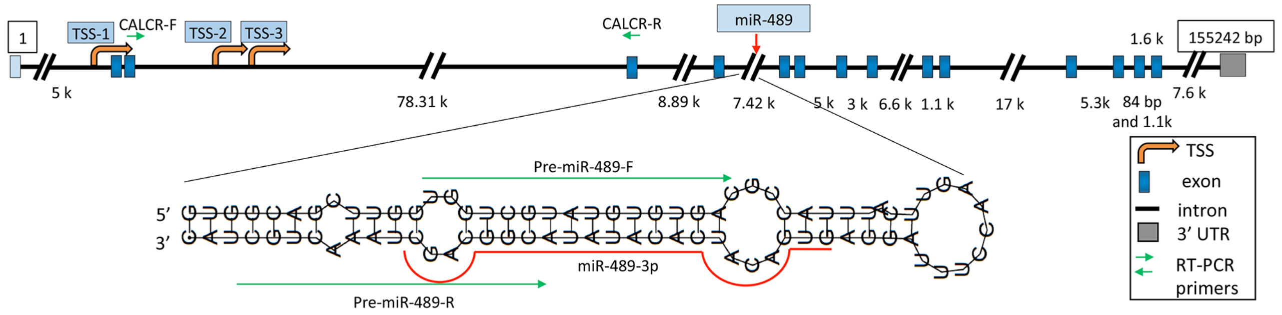

Supplementary Figure S1: Schematic representation of calcitonin receptor gene showing location of miR-489. The schematic showing ~155kb long Calcitonin receptor (CALCR) gene, which is located on the chromosome 7. CALCR gene is complex and has numerous introns. Pre-miR-489 is located in the intron of CALCR gene. Primer locations for CALCR and pre-miR-489 are shown with green arrows.

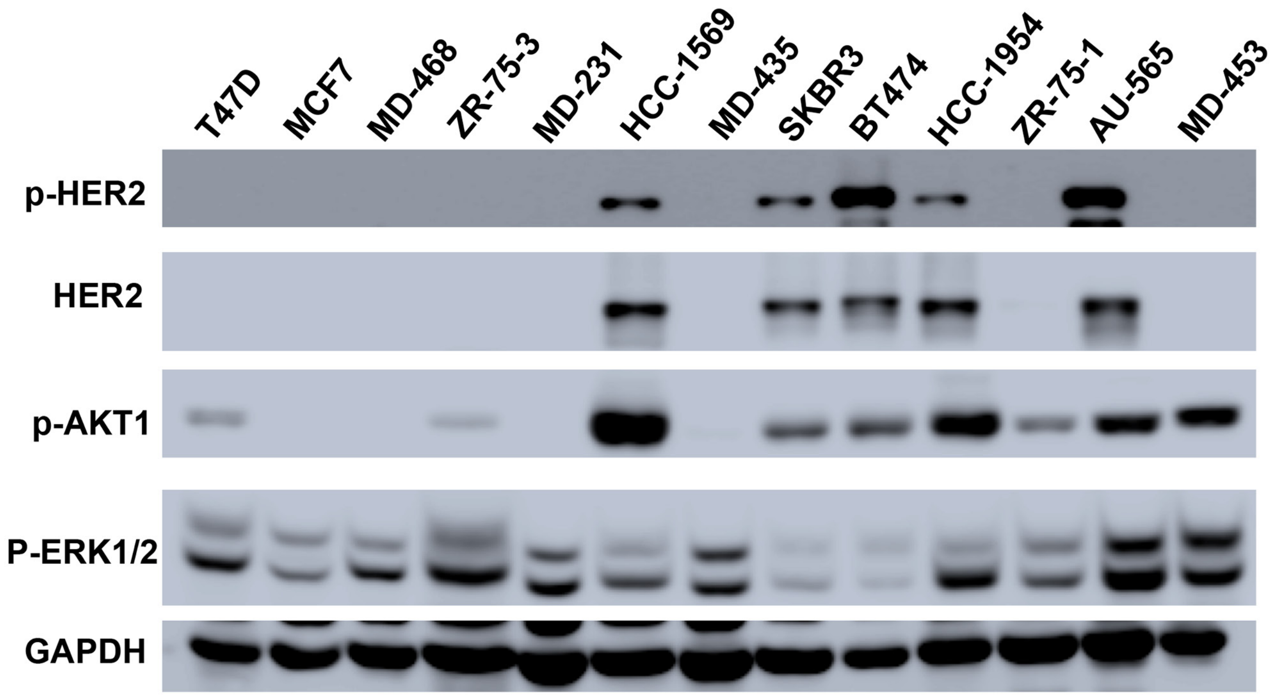

Supplementary Figure S2: Western blot analysis of HER2-signaling pathways in breast cancer cell lines.

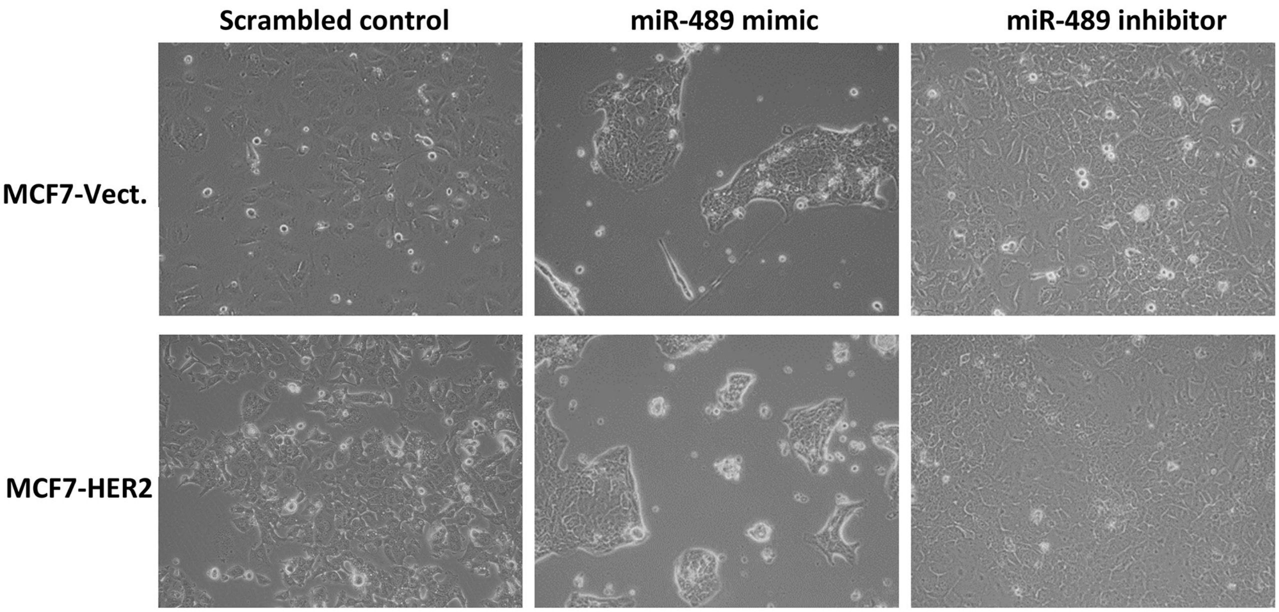

Supplementary Figure S3: Effect of mimic-miR-489 and inhibitor-miR-489 on the growth and morphology of MCF-7 vect and HER2 cells. MCF7 vect and HER2 cells were treated with mimic-miR-489 or inh.-miR-489 as mentioned in the methods for 72 h. Representative images of each cell line for each condition was captured using light microscopy at 10x magnification.

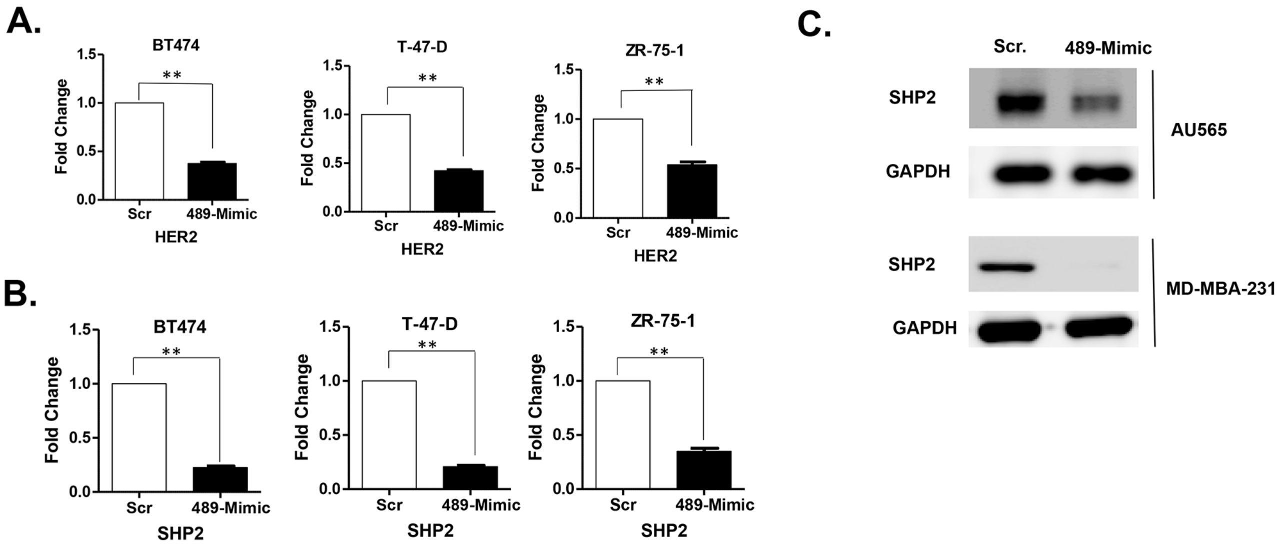

Supplementary Figure S4: Down regulation of SHP2 and HER2 at protein and mRNA level upon transfection with miR-489-mimic. A and **B.** qRT-PCR analysis of SHP2 and HER2 mRNA expression in breast cancer cell lines transfected with miR-489 mimic or mock (**$p < 0.01$). **C.** Western blot analysis of other breast cancer cell lines treated with miR-489 mimic also showed reduction in SHP2 expression

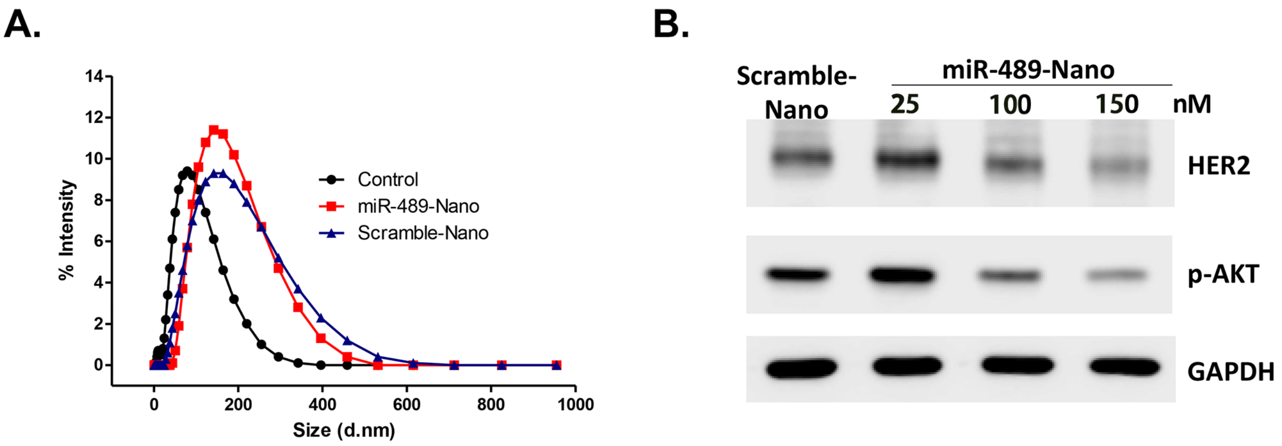

Supplementary Figure S5: Synthesis and characterization of miR-489-delivery nanoparticles. A. Size determination of nanoparticle alone or packed with either scramble miR or mimic miR-489 using dynamic light scattering. The average size of nanoparticles packed with scramble or mimic do not vary significantly. **B.** HCC1954 Cells were treated with increasing concentration of mimic-489 packed in nano particles for 72 hr. Expression of HER2 was suppressed with increasing concentration of miR-489.

Supplementary Table S1: Expression analysis of different miRNAs in MCF7 HER2 cells compared to the MCF7 Vect cells.

See Supplementary File 1

Supplementary Table S2: The clinicopathological characteristics of clinical samples

Biopsy no	Histopathological description	Grade	ER	PR	HER2
08-9111	Invasive ductal carcinoma	3	0	0	3
26194	Invasive ductal carcinoma	3	1	1	1
31496	Papillary carcinoma	3	0	0	0
25173	Invasive ductal carcinoma	3	1	1	1
31924	Invasive ductal carcinoma	2	1	1	0
31423	Invasive ductal carcinoma	2	1	1	2
31926	Invasive ductal carcinoma	3	0	0	1
40104	Invasive ductal carcinoma	3	0	0	NA
40401	Paget disease and invasive ductal carcinoma	2	1	1	NA
40172	Invasive ductal carcinoma	3	0	0	0
31870	Invasive ductal carcinoma	3	0	0	3
40604	Invasive ductal and lobular carcinoma	2	1	1	0

Supplementary Table S3: The correlation of miR-489 expression with other prognostic factors for breast cancer

Characteristics	Total (n=868)	miR-489 low (n= 434)		miR-489 high (n= 434)		P value*
		No.	%	No.	%	
**HER2 Status**						
Positive	111	68	61.3	43	38.7	0.011
Negative	757	366	48.3	391	51.6	
**Grade**						
I-II	396	131	33.1	265	66.9	< 0.0001
III	428	282	65.9	146	34.1	
missing	45	22	48.9	23	51.1	
**Distant Metastasis**						
yes	225	130	57.8	95	42.2	0.0067
no	645	305	47.3	340	52.7	
**Tumor size**						
<=2 cm	376	160	42.5	216	57.4	0.0012
>2cm	492	274	55.7	218	44.3	
**Stages**						
0-I	279	113	40.5	166	59.5	0.0005
II	401	210	52.4	191	47.6	
III-IV	73	46	63.0	27	37.0	
missing	112	63	56.3	49	43.7	

Note: *, the differential expression of miR-489 among different subgroups were analyzed using Chi square test. Values of P < 0.05 were considered significant.
