# Supplementary material for: A novel double-negative feedback loop between miR-489 and the HER2-SHP2-MAPK signaling axis regulates breast cancer cell proliferation and tumor growth
Source: Oncotarget. 2016 Feb 22;7(14):18295–308. doi: 10.18632/oncotarget.7577 (PMC4951289; doi:10.18632/oncotarget.7577)
Supplement: Supplementary file 2 [file oncotarget-07-18295-s002.docx]

**Supplementary Tables**

**Table S1. Expression analysis of different miRNAs in MCF7 HER2 cells compared to the MCF7 Vect cells**

| miRNA Name | Experiment #1 |  |  | Experiment #2 |  |  | Exp. Ratio (HER2/Vect) | SD |
| --- | --- | --- | --- | --- | --- | --- | --- | --- |
|  | Vect ΔCt | HER2 ΔCt | ΔΔCt | Vect ΔCt | HER2 ΔCt | ΔΔCt |  |  |
| 135b-5P | 14.47 | 9.48 | 4.99 | 17.14 | 13.02 | 4.12 | 24.6 | 10.15 |
| 184 | 15.51 | 11.49 | 4.02 | 15.92 | 12.85 | 3.07 | 12.3 | 5.54 |
| 10a-5P | 10.4 | 7.51 | 2.89 | 15.35 | 11.64 | 3.71 | 10.23 | 4.01 |
| 219-5p | 15.11 | 11.96 | 3.15 | 15.83 | 13.48 | 2.35 | 6.99 | 2.7 |
| 32-5p | 9.97 | 7.17 | 2.8 | 10.36 | 9.49 | 0.87 | 4.4 | 3.65 |
| Let 7a-5P | 7.7 | 6.67 | 1.03 | 10.14 | 7.49 | 2.65 | 4.16 | 3.01 |
| 329 | 16.37 | 13.73 | 2.64 | 13.15 | 13.81 | -0.66 | 3.43 | 3.95 |
| 33b-5p | 7.41 | 5.98 | 1.43 | 8.95 | 6.91 | 2.04 | 3.4 | 1 |
| let 7g-5P | 7.57 | 6.63 | 0.95 | 8.78 | 6.95 | 1.83 | 2.74 | 1.15 |
| 200b-3p | 4.71 | 3.67 | 1.04 | 11.88 | 10.22 | 1.66 | 2.61 | 0.78 |
| 449a | 12.65 | 11.89 | 0.76 | 13.24 | 11.49 | 1.75 | 2.53 | 1.19 |
| 365a-3p | 9.02 | 8.66 | 0.35 | 10.31 | 8.43 | 1.87 | 2.47 | 1.69 |
| 200a-3p | 4.77 | 4.07 | 0.71 | 7.38 | 5.75 | 1.63 | 2.37 | 1.04 |
| 29c-3p | 5.5 | 4.72 | 0.77 | 6.92 | 5.37 | 1.54 | 2.31 | 0.85 |
| 141-3p | 2.63 | 2.33 | 0.3 | 7.01 | 5.34 | 1.68 | 2.22 | 1.39 |
| let 7e-5P | 4.71 | 4.34 | 0.38 | 8.27 | 6.64 | 1.63 | 2.19 | 1.26 |
| 320c | 7.35 | 6.24 | 1.11 | 10.53 | 9.4 | 1.13 | 2.17 | 0.02 |
| 21-5p | -3.12 | -4.06 | 0.94 | -0.78 | -1.96 | 1.19 | 2.1 | 0.25 |
| 7-5p | 5.71 | 5.3 | 0.41 | 10.21 | 8.73 | 1.47 | 2.05 | 1.03 |
| 30e-5p | 9.41 | 8.17 | 1.24 | 11.03 | 10.32 | 0.72 | 2.01 | 0.51 |
| 223 | 10.93 | 10.91 | 0.02 | 14.54 | 13.02 | 1.51 | 1.94 | 1.3 |
| 425-5p | 1.91 | 1.77 | 0.13 | 6.71 | 5.32 | 1.39 | 1.86 | 1.08 |
| 222-3p | 6.16 | 5.36 | 0.8 | 6.41 | 5.45 | 0.97 | 1.85 | 0.15 |
| 9-5p | 11.7 | 12.55 | -0.85 | 14.92 | 13.29 | 1.63 | 1.82 | 1.79 |
| 221-3p | 4.55 | 4.16 | 0.39 | 4.4 | 3.18 | 1.22 | 1.82 | 0.72 |
| 539-5p | 15.41 | 15.65 | -0.24 | 19.56 | 18.08 | 1.47 | 1.81 | 1.37 |
| 208b | 14.82 | 13.17 | 1.65 | 15.22 | 16.39 | -1.17 | 1.79 | 1.9 |
| 139-5P | 7.25 | 6.54 | 0.71 | 7.69 | 6.98 | 0.72 | 1.64 | 0.01 |
| 320b | 1.71 | 1.02 | 0.69 | 1.78 | 1.12 | 0.66 | 1.6 | 0.02 |
| 29b-3p | 6.86 | 6.36 | 0.51 | 9.78 | 8.97 | 0.82 | 1.59 | 0.24 |
| 106b-5P | 1.6 | 0.98 | 0.62 | 2.6 | 1.9 | 0.69 | 1.58 | 0.06 |
| 29a-3p | 4.87 | 4.85 | 0.02 | 5.13 | 4.05 | 1.08 | 1.56 | 0.78 |
| 378a-3p | 3.16 | 2.78 | 0.38 | 3.3 | 2.47 | 0.83 | 1.54 | 0.34 |
| 193a-5p | 3.46 | 3.48 | -0.01 | 4.71 | 3.65 | 1.07 | 1.54 | 0.78 |
| 369-5p | 14.51 | 13.37 | 1.14 | 19.54 | 19.76 | -0.22 | 1.53 | 0.96 |
| 25-3p | -1.52 | -1.74 | 0.22 | -0.78 | -1.7 | 0.92 | 1.53 | 0.52 |
| 215 | 7.68 | 7.66 | 0.02 | 9.53 | 8.51 | 1.03 | 1.52 | 0.72 |
| 141 | 2.34 | 2.43 | -0.09 | 4.72 | 3.68 | 1.05 | 1.5 | 0.79 |
| 33a-5p | 2.95 | 2.29 | 0.65 | 3.23 | 2.8 | 0.43 | 1.46 | 0.16 |
| 26a-5p | 1.3 | 1.46 | -0.16 | 4.27 | 3.27 | 1.01 | 1.45 | 0.79 |
| 30b-5p | 2.45 | 1.79 | 0.66 | 3.51 | 3.13 | 0.38 | 1.44 | 0.2 |
| 320a | -1.09 | -1.66 | 0.56 | -0.73 | -1.21 | 0.48 | 1.44 | 0.06 |
| 296-5p | 1.83 | 1.59 | 0.23 | 1.83 | 1.07 | 0.76 | 1.44 | 0.37 |
| 218-5p | 11.75 | 10.86 | 0.89 | 13.38 | 13.43 | -0.05 | 1.41 | 0.63 |
| 362-5p | 8.01 | 7.15 | 0.86 | 7.49 | 7.56 | -0.07 | 1.38 | 0.61 |
| 455-5p | 7.19 | 6.82 | 0.38 | 7.2 | 6.68 | 0.52 | 1.37 | 0.1 |
| 22-3p | 2.24 | 2.04 | 0.21 | 1.93 | 1.3 | 0.63 | 1.35 | 0.28 |
| 30a-5p | 7.8 | 7.24 | 0.56 | 9.48 | 9.19 | 0.29 | 1.35 | 0.18 |
| 429 | 5.11 | 4.72 | 0.39 | 8.08 | 7.63 | 0.45 | 1.34 | 0.04 |
| 200c-3p | -2.09 | -2 | -0.09 | -0.31 | -1.1 | 0.79 | 1.34 | 0.56 |
| 365b-3p | 7.64 | 8.8 | -1.16 | 9.41 | 8.26 | 1.14 | 1.33 | 1.25 |
| 30c-5p | 3.16 | 2.55 | 0.61 | 3.82 | 3.68 | 0.14 | 1.31 | 0.3 |
| 194-5p | 3.22 | 3.19 | 0.03 | 4.01 | 3.35 | 0.65 | 1.3 | 0.39 |
| 95 | 8.17 | 8.9 | -0.73 | 14.22 | 13.23 | 0.99 | 1.3 | 0.98 |
| 338-5p | 6.54 | 6.13 | 0.4 | 7.92 | 7.61 | 0.31 | 1.28 | 0.06 |
| 501-5p | 9.77 | 9.34 | 0.42 | 9.79 | 9.55 | 0.24 | 1.26 | 0.12 |
| 205 | 5.97 | 6.21 | -0.23 | 7.6 | 6.88 | 0.73 | 1.25 | 0.57 |
| let 7c | 6.67 | 6.8 | -0.13 | 10.36 | 9.69 | 0.66 | 1.25 | 0.48 |
| 19a-3p | 0.36 | 0.81 | -0.45 | 2.55 | 1.73 | 0.81 | 1.25 | 0.73 |
| 504 | 8.2 | 8.31 | -0.1 | 10.37 | 9.8 | 0.56 | 1.2 | 0.39 |
| 301c-3p | 0.46 | 0.61 | -0.15 | 2.1 | 1.56 | 0.53 | 1.17 | 0.39 |
| 34a-5p | 0.71 | 0.44 | 0.27 | 0.4 | 0.21 | 0.18 | 1.17 | 0.05 |
| 199a-3p | 11.48 | 11.68 | -0.2 | 12.67 | 12.12 | 0.55 | 1.17 | 0.42 |
| 193b-3p | -0.73 | -0.52 | -0.2 | -0.54 | -1.07 | 0.53 | 1.16 | 0.41 |
| 30d-5p | 3.27 | 3.14 | 0.13 | 4.74 | 4.46 | 0.28 | 1.15 | 0.08 |
| 93-5p | -1.1 | -1.21 | 0.11 | -0.73 | -1.01 | 0.28 | 1.15 | 0.09 |
| 103a-3P | -1.07 | -1.31 | 0.24 | -0.83 | -0.96 | 0.13 | 1.14 | 0.06 |
| 212 | 5.41 | 5.78 | -0.37 | 6.53 | 5.95 | 0.58 | 1.14 | 0.51 |
| 150-5p | 12.02 | 11.63 | 0.39 | 12.07 | 12.16 | -0.09 | 1.13 | 0.26 |
| let 7i -5P | 2.49 | 2.21 | 0.28 | 3.19 | 3.14 | 0.05 | 1.12 | 0.13 |
| 361-5p | 8.74 | 8.74 | -0.01 | 13.84 | 13.52 | 0.31 | 1.12 | 0.18 |
| 302c-3p | 17.62 | 19.16 | -1.54 | 18.67 | 17.76 | 0.91 | 1.11 | 1.09 |
| let 7d-5P | 2.24 | 2.27 | -0.03 | 3.96 | 3.67 | 0.29 | 1.1 | 0.17 |
| 181b-5p | 4.7 | 4.25 | 0.45 | 4.56 | 4.94 | -0.38 | 1.07 | 0.42 |
| 192-5p | 3.43 | 3.07 | 0.36 | 4.44 | 4.67 | -0.24 | 1.07 | 0.31 |
| 126-3P | 1.84 | 1.59 | 0.25 | 2.7 | 2.81 | -0.11 | 1.06 | 0.18 |
| 146a-5P | 9.9 | 11.12 | -1.21 | 14.01 | 13.27 | 0.74 | 1.05 | 0.88 |
| 205-5p | 4.6 | 5.02 | -0.43 | 5.08 | 4.66 | 0.42 | 1.04 | 0.42 |
| 422a | 5.73 | 6.23 | -0.5 | 6.6 | 6.15 | 0.46 | 1.04 | 0.47 |
| 181d | 5.51 | 5.22 | 0.3 | 5.73 | 6 | -0.27 | 1.03 | 0.28 |
| 500a-5p | 7.69 | 7.79 | -0.1 | 8.71 | 8.54 | 0.17 | 1.03 | 0.13 |
| 23a-3p | -2.29 | -1.81 | -0.48 | -1.15 | -1.55 | 0.4 | 1.02 | 0.43 |
| 326 | 4.3 | 4.73 | -0.44 | 4.5 | 4.13 | 0.37 | 1.02 | 0.39 |
| 125a-5P | -1.45 | -1.4 | -0.04 | -0.08 | -0.14 | 0.05 | 1 | 0.05 |
| 193a-5p | 3.73 | 4.16 | -0.44 | 4.58 | 4.27 | 0.31 | 0.99 | 0.35 |
| 199b-5p | 7.44 | 7.86 | -0.43 | 9.13 | 8.83 | 0.3 | 0.99 | 0.35 |
| 191-5p | -0.56 | -0.67 | 0.11 | 0.39 | 0.58 | -0.19 | 0.98 | 0.14 |
| 485-5p | 12.21 | 12.77 | -0.56 | 13.37 | 13.04 | 0.33 | 0.97 | 0.41 |
| 500b | 15.74 | 15.37 | 0.37 | 17.44 | 18.1 | -0.66 | 0.96 | 0.47 |
| 523-3p | 13.56 | 14.15 | -0.59 | 13.18 | 12.85 | 0.33 | 0.96 | 0.42 |
| 96-5p | 2.58 | 2.97 | -0.39 | 3.65 | 3.48 | 0.17 | 0.94 | 0.26 |
| 20b-5p | 6.38 | 6.67 | -0.3 | 6.82 | 6.72 | 0.1 | 0.94 | 0.18 |
| 196b-5p | 6.73 | 7.56 | -0.83 | 8.32 | 7.93 | 0.4 | 0.94 | 0.53 |
| 99b-5p | -1.14 | -0.79 | -0.35 | -0.81 | -0.92 | 0.11 | 0.93 | 0.21 |
| 495-3p | 12.93 | 12.31 | 0.62 | 12.36 | 14.06 | -1.7 | 0.92 | 0.87 |
| 301b | 4.44 | 4.65 | -0.2 | 5.22 | 5.26 | -0.04 | 0.92 | 0.08 |
| 19b-3p | -0.58 | -0.08 | -0.51 | 0.34 | 0.16 | 0.19 | 0.92 | 0.31 |
| 339-5p | -0.66 | -0.66 | 0 | -0.51 | -0.24 | -0.27 | 0.91 | 0.12 |
| 155-5p | 17.08 | 16.52 | 0.56 | 19.26 | 20.94 | -1.68 | 0.9 | 0.82 |
| 146b-5p | 5.77 | 5.75 | 0.02 | 7.09 | 7.47 | -0.38 | 0.89 | 0.18 |
| 148a-3p | 3.5 | 3.41 | 0.09 | 3.82 | 4.43 | -0.61 | 0.86 | 0.29 |
| 203a | 3.77 | 5.53 | -1.76 | 7.2 | 6.7 | 0.5 | 0.86 | 0.79 |
| 151a-5p | 0.17 | 0.32 | -0.15 | 0.42 | 0.74 | -0.33 | 0.85 | 0.07 |
| 132-3P | 4.14 | 4.35 | -0.21 | 4.91 | 5.2 | -0.29 | 0.84 | 0.03 |
| 299-5p | 12.57 | 15.86 | -3.29 | 15.97 | 15.31 | 0.66 | 0.84 | 1.05 |
| 34c-3p | 11.76 | 11.37 | 0.4 | 13.26 | 14.72 | -1.46 | 0.84 | 0.67 |
| 130b-3P | 5.56 | 5.96 | -0.4 | 5.69 | 5.84 | -0.15 | 0.83 | 0.1 |
| Let 7b-5P | 2.68 | 2.88 | -0.2 | 2.74 | 3.1 | -0.35 | 0.83 | 0.06 |
| 18b-5p | 7.18 | 8.34 | -1.16 | 10.6 | 10.36 | 0.24 | 0.82 | 0.52 |
| 212-3p | 8.69 | 8.92 | -0.23 | 13.45 | 13.84 | -0.39 | 0.81 | 0.06 |
| 140-5P | 7.6 | 7.52 | 0.07 | 9.37 | 10.23 | -0.87 | 0.8 | 0.36 |
| 152 | 2.73 | 2.94 | -0.21 | 3.48 | 3.94 | -0.45 | 0.8 | 0.09 |
| 505-3p | 3.77 | 3.99 | -0.22 | 4.41 | 4.85 | -0.44 | 0.8 | 0.09 |
| 526b-5p | 14.09 | 14.55 | -0.46 | 18.04 | 18.25 | -0.21 | 0.8 | 0.1 |
| 153 | 15.92 | 15.87 | 0.04 | 16.56 | 17.43 | -0.87 | 0.79 | 0.34 |
| 335-5p | 9.72 | 9.65 | 0.07 | 13.06 | 13.99 | -0.93 | 0.79 | 0.37 |
| 16-5p | -3.58 | -3.43 | -0.15 | -2.9 | -2.32 | -0.59 | 0.78 | 0.17 |
| 15a-5p | 0.58 | 0.62 | -0.04 | 0.63 | 1.39 | -0.76 | 0.78 | 0.27 |
| 148b-3p | 2.98 | 3.34 | -0.37 | 3.32 | 3.66 | -0.34 | 0.78 | 0.01 |
| 24-3p | -0.71 | 0.05 | -0.76 | -0.21 | -0.15 | -0.05 | 0.78 | 0.26 |
| 23b-3p | -0.66 | -0.15 | -0.51 | -0.24 | 0.05 | -0.29 | 0.76 | 0.08 |
| 377-3p | 11.39 | 12.92 | -1.53 | 13.73 | 13.51 | 0.23 | 0.76 | 0.58 |
| 331-5p | 9.17 | 9.1 | 0.08 | 8.66 | 9.83 | -1.17 | 0.75 | 0.43 |
| 20a-5p | 1.51 | 2.01 | -0.5 | 2.07 | 2.42 | -0.35 | 0.75 | 0.05 |
| 149-5p | 1.64 | 2.13 | -0.48 | 2.83 | 3.23 | -0.4 | 0.74 | 0.03 |
| 342-5p | 8.41 | 9.21 | -0.8 | 8.62 | 8.78 | -0.16 | 0.73 | 0.23 |
